# Using Artificial Intelligence to Develop Clinical Decision Support Systems—The Evolving Road of Personalized Oncologic Therapy

**DOI:** 10.3390/diagnostics15182391

**Published:** 2025-09-19

**Authors:** Elena Chitoran, Vlad Rotaru, Aisa Gelal, Sinziana-Octavia Ionescu, Giuseppe Gullo, Daniela-Cristina Stefan, Laurentiu Simion

**Affiliations:** 1 Medicine School, “Carol Davila” University of Medicine and Pharmacy, 050474 Bucharest, Romania; 2 General Surgery and Surgical Oncology Department I, Bucharest Institute of Oncology “Prof. Dr. Alexandru Trestioreanu”, 022328 Bucharest, Romania; 3 Department of Obstetrics and Gynecology, Villa Sofia Cervello Hospital, University of Palermo, 90146 Palermo, Italy

**Keywords:** machine learning, artificial intelligence, clinical score, personalized treatment, targeted therapy, clinical tools, interactive forms, clinical decision support system

## Abstract

**Background/Objectives:** The use of artificial intelligence (AI) in oncology has the potential to improve decision making, particularly in managing the risk associated with targeted therapies. This study aimed to develop and validate a machine learning-based clinical decision support system (CDSS) capable of predicting complications associated with Bevacizumab or its biosimilars and to translate the resulting predictive model into a clinically applicable tool. **Methods:** A prospective observational study was conducted on 395 records from patients treated with Bevacizumab or biosimilars for solid tumors. Pretherapeutic variables, such as demographic data, medical history, tumor characteristics and laboratory findings, were retrieved from medical records. Several machine learning models (logistic regression, Random Forest, XGBoost) were trained using 70/30 and 80/20 data splits. Their predictive performances were compared using accuracy, AUC-ROC, sensitivity, specificity, F1-scores and error rate. The best-performing model was used to derive a logistic-based risk score, which was further implemented as an interactive HTML form. **Results:** The optimized Random Forest model trained on the 80/20 split demonstrated the best balance between accuracy (70.63%), sensitivity (66.67%), specificity (73.85%), and AUC-ROC (0.75). The derived logistic risk score showed good performance (AUC-ROC = 0.720) and calibration. It identified variables, such as age ≥ 65, anemia, elevated urea, leukocytosis, tumor differentiation, and stage, as significant predictors of complications. The final tool provides clinicians with an easy-to-use, offline form that estimates individual risk levels and stratifies patients into low-, intermediate-, or high-risk categories. **Conclusions:** This study offers a proof of concept for developing AI-supported predictive tools in oncology using real-world data. The resulting logistic risk score and interactive form can assist clinicians in tailoring therapeutic decisions for patients receiving targeted therapies, enhancing the personalization of care without replacing clinical judgment.

## 1. Introduction

In the last few years, oncological treatment has rapidly evolved and targeted therapies have become readily available for a large number of patients on a global scale. As such, oncology has generated an ever-increasing influx of real-time data. The development of artificial intelligence tools provides an enhanced possibility of data synthesis and opens the way for innovative perspectives in creating advanced clinical tools. These tools (or clinical decision support systems—CDSSs) are not meant to replace medical expertise but to serve as complementary tools and help improve the accuracy and speed of the decisional process [1].

Artificial intelligence (AI) tools can be used to rapidly process high volumes of, often, disjointed data (clinical, paraclinical, imaging, molecular, genetic) and extract predictive patterns difficult to observe through traditional methods, which can be used for developing clinical scores. Interactive forms based on AI algorithms can be used to evaluate the patient’s risk of developing complications of targeted therapies in real time, such as angiogenesis inhibitors. CDSSs can be used during the consultation to guide the discussion with the patient about therapeutic alternatives, possible risks and expected benefits, contributing to a shared medical decision. They can integrate multiple variables—from age and comorbidities to biomarkers and functional scores—to generate a personalized risk assessment [2]. Integrating these tools into current oncology practice offers the opportunity to optimize the risk–benefit ratio of therapies, especially in complex or marginal cases. For example, in elderly patients with multiple comorbidities, AI-based CDSSs (AI-CDSSs) can suggest an increased likelihood of severe toxicity with a particular therapy, thus guiding the medical team towards better-tolerated alternatives. At the same time, these systems can alert the clinician to the need for stricter paraclinical monitoring or dose adjustment but do not replace clinical judgment or therapeutic guidelines [3,4].

Another great advantage of AI-CDSSs is their ability for continuous and dynamic updates, by adding new data as they become available through clinical observation in real oncological practice. The clinical scores can be continuously refined through feed-back and this aspect is especially essential in oncology. As real-life data accumulate, AI models can be automatically recalibrated. This allows for continuous improvements to scores and forms, aligning them with evolving therapies and real-world outcomes in the population [5,6]. It is, however, essential that these tools are rigorously validated in real clinical settings, through multidisciplinary collaboration between oncologists, biostatisticians, AI experts and medical informaticians.

But the integration of AI technology in clinical practice needs to be made with responsibility. Algorithmic bias must be considered—AI models may underestimate the risk of certain groups or misinterpret clinical variables, requiring rigorous validation and representative inclusion of data [7]. No matter how performant the AI-CDSSs are [8], they cannot replace detailed clinical exams, direct discussions with the patients and a correct evaluation of each patient’s medical context. Also, international guidelines, which are based on solid clinical evidence, need to remain the basis of any therapeutic decision [9].

The CDSS interface must be intuitive, integrable into electronic health systems, and comply with patient data protection standards. Their acceptability among professionals also depends on the transparency of the algorithms and their ability to provide clear explanations for the recommendations made. A recent study showed an increased level of physician trust in the AI tool when the results of the AI tool concord with the results of randomized controlled trails [10], thus raising the problems of transparency and model explainability. Moreover, the success of AI-CDSSs in oncology is dependent on the quality of available data [11].

Recent advances in artificial intelligence (AI) and machine learning (ML) have led to highly performant clinical decision support systems (CDSSs). However, most of these models are developed in collaboration with IT experts, often resulting in complex black-box systems that are difficult for clinicians to understand and apply in daily practice. As a result, a gap remains between the potential of AI technologies and their adoption in oncology. Our work addresses this gap by presenting a clinician-led proof of concept. The goal was not to design the most accurate or complex predictive model but to demonstrate that contemporary AI tools have become sufficiently user-friendly to be directly applied by physicians without advanced computational skills. In this way, we aim to reduce clinicians’ apprehension toward AI, to show feasibility, and to bridge the professional distance between medicine and computer science.

The aim of this study is to offer a proof of concept on how AI tools can be used for developing clinical interactive and predictive tools, which may in fact represent a major step to truly personalized oncological care, while simultaneously proving that these kinds of tools are now so readily available and intuitive that clinicians with no formal computational training can integrate them into both research and clinical practice with promising results. At the same time, we emphasize that interdisciplinary collaboration with AI specialists is crucial for model enhancement, performance, interpretability and clinical translation.

The main contributions of this study can be summarized as follows:

- We developed a clinician-led, proof-of-concept CDSS for predicting complications of Bevacizumab using routinely available pretherapeutic data.
- We tested several machine learning algorithms (logistic regression, Random Forest, XGBoost) and selected the best-performing approach.
- We derived a simplified logistic regression-based risk score and translated it into an accessible offline HTML calculator for clinical use.
- We demonstrated feasibility by applying user-friendly AI tools to a real-world oncology dataset without advanced IT expertise.
- We positioned this work not as a final model but as a step toward reducing clinicians’ apprehension toward AI and fostering future multidisciplinary collaborations.

## 2. Materials and Methods

### 2.1. Patient Selection and Data Collection

This study was conducted in accordance with the Declaration of Helsinki and approved by the Institutional Ethics Committee of Bucharest Institute of Oncology “Prof. Dr. Alexandru Trestioreanu” (Protocol Number. 24855, Date of approval: 24 November 2022). The design of this study was open, observational, prospective, conducted under real conditions of current oncological practice on patients treated with Bevacizumab or biosimilars, anticancer drugs associated with quite frequent and sometimes serious side effects [12,13]. We included all patients treated for solid malignant tumors at the Bucharest Oncological Institute “Prof. Dr. Al. Trestioreanu (IOB) between 1 January 2023 and 30 June 2024, consecutively. This research was intended to evaluate the usual clinical practice and the real experience of using this drug, which is why the data were extracted from the patients’ medical records and from the hospital’s electronic database. We did not specify exclusion criteria, and all medical decisions regarding treatment, doses, regimens or follow-up were made by the attending physicians without any limitation or influence. We extended the patient follow-up period until 31 December 2024, when the patients were censored prior to the analysis. Thus, we ensured a minimum follow-up period of 6 months.

All patients admitted in IOB agree, by signing a consent form, to the anonymous usage of their medical records and investigation results for scientific, educational and for the preparation and publication of scientific papers, with no additional consent required unless the patient has withdrawn consent.

Patients who received Bevacizumab or biosimilars were identified from pharmacy consumption tables. If a patient received more than one line of therapy containing Bevacizumab, each line of treatment was considered a separate record and analyzed as such. Similarly, if a patient received Bevacizumab for multiple neoplastic diseases, each indication was considered a separate record.

Case-specific data were obtained from patient charts, electronic records, and by consulting laboratory, imaging, histopathological, and genetic results. Data collected included demographics, comorbidities and relevant medical history, neoplastic disease history (tumor origin, histology, immunohistochemistry, genetic characteristics, metastatic sites, diagnostic dates, treatment initiation, progression, death), treatment aspects (line of treatment, combination therapies, dosage, duration of treatment, adverse events, treatment response).

Complications were defined as any documented adverse event considered at least possibly related to Bevacizumab or its biosimilars, regardless of severity, as recorded in patient charts and according to clinical judgment. We initially aimed to model only severe complications, but due to their low incidence, the dataset was statistically underpowered for such stratification. We, thus, included all complications as a binary outcome, which reflects a real-world setting where even moderate events may require dose adjustments or impact quality of life.

### 2.2. Variable Selection

We used several predictive models developed through machine learning to estimate the risk of complications associated with Bevacizumab, based on pretherapeutic variables, including the following: socio-demographic data of the patients (such as age, sex, background, health insurance status), the presence of personal history (smoking, alcohol, nutritional status), the presence of pathological history (ischemic heart disease, hypertension, heart failure, arrhythmias, valvular diseases, diabetes mellitus, previous treatment with oral anticoagulants/oral antidiabetics/insulin, chronic renal or pulmonary disease, stroke/TIA, thromboembolic events, chronic hepatitis or cirrhosis, other neoplasia than the one being treated, psychiatric history, recurrent urinary tract infections, surgical history), data on the neoplastic disease (TNM classification, location of metastases, stage of the disease, histopathological form, degree of differentiation, neoplastic infiltration of the margins of the resection, presence of lymphovascular or perineural invasion and presence of peritumoral lymphocytic infiltrate on resection pieces/biopsy samples), indication and method of administration of Bevacizumab (unresectable locally advanced neoplastic disease/metastatic disease/recurrent disease, therapeutic line 1/2/3+/maintenance), history of oncological treatments (chemo-radiotherapy, hormone-, immunotherapy, oncological surgical interventions), pretherapeutic biological parameters (hemoglobin, leukocytes, platelets, blood glucose, urea and creatinine, serum Ca^2+^, transaminases, GGT, presence of coagulation changes or tumor markers) and quality of life of patients prior to initiation of therapy. Unfortunately, although we tried to collect data on tumor receptors, the presence of oncogenic mutations or deficiencies in repair mechanisms, these data could not be used to generate predictive models even when we tried to merge them, due to the massive percentage of missing data (over 80% of patient records do not contain such data, which indicates a lack of accessibility for most patients to advanced genetic testing methods, which may influence their ability to benefit from truly targeted therapies).

### 2.3. Database Pre-Processing and Leakage Prevention

In an attempt to achieve this objective, we processed the initial database by eliminating all variables that described the type of complications that occurred and their consequences, maintaining only the pretherapeutic characteristics of the patients and diseases that in theory could serve as predictive factors for the occurrence of complications. Any post-treatment or outcome-linked fields were removed a priori to avoid information leakage. Missing values were imputed within the training data only and the fitted imputers were retrained inside each cross-validation fold before being applied to the corresponding validation fold. No outcome-aware univariate filtering or feature selection was performed outside the cross-validation loop.

We also added to the database a recoded binary variable with possible values 0 for the absence of any Bevacizumab-associated complication and 1 for the occurrence of at least one therapy-associated complication regardless of its severity. We did not attempt to create predictive models for severe complications, because the number of cases where they occurred is relatively small, which may lead to a decrease in the predictive power of the models.

A rigorous cleaning of the database was performed in several successive steps, in order to optimize it. Initially, we identified variables that contained a significant number of missing values. Thus, variables encoding certain biological constants such as Na^+^, K^+^ and Cl^−^ (frequently determined together but absent in over 20% of the records prior to the initiation of therapy) were completely eliminated from the analysis to avoid introducing errors in the imputation process (automatic completion) of the data. Subsequently, for continuous numerical variables with a small number of missing values (<5%), we opted for an imputation method based on the median value of the entire sample—applied to the variables encoding GGT and serum Ca^2+^. The last step we performed to clean the dataset was an automated analysis of the mean, minimum and maximum values of the continuous numeric variables that highlighted possible outliers. This identified 2 data entry errors (omitting a comma) that were manually corrected after rechecking the values in the patient records.

### 2.4. Training Predictive Models

We used the optimized database to train several predictive machine learning models:

- Simple logistic regression—model based on linear relationships between predictors and log-odds, relatively sensitive to outliers and noisy variables, but which offers the advantage of obtaining coefficients that are easily interpretable in a clinical context.
- Penalized logistic regression with Elastic Net—method that combines the advantages of L1 (sparse, variable selection) and L2 (stability) regularization.
- Random Forest—robust nonlinear model, based on decision trees, with great capacity to capture complex relationships.
- XGBoost—boosting-type model (improves errors gradually) that offers the highest accuracy, superior performance on complicated data and good management of imbalances but at the cost of lower interpretability.

Short, clinician-oriented “algorithm primers” summarizing working principles, strengths, and limitations for Logistic/Elastic-Net, Random Forest, and XGBoost are available in previous works [14].

To mitigate moderate class imbalance, we applied class weights (inverse frequency) for Logistic/Elastic-Net and balanced class weights for RF and XGBoost during training. Unless otherwise stated, metrics were computed at a default 0.50 probability threshold; alternative thresholds can trade sensitivity for specificity depending on clinical priorities.

Statistical analysis and development of predictive models were performed using a cloud-based interactive web platform, Jupyter notebook (Google Colab [15]). The Python scripts used in the modeling process were developed with the help of web-based AI code generation assistants available on the platform and online (Gemini 2.0 model [15] and ChatGPT plus 4o [16]), used exclusively under active human supervision and guided by the requirements formulated by the authors. These tools were also used for figure generation. All analytical and methodological decisions, interpretations and scientific writing were made exclusively by the authors.

In the process of developing the predictive models, datasets were initially divided in a proportion of 70% for training the model and 30% for testing, a common choice that provides a good balance between learning and evaluation. However, to improve the models’ learning ability (especially in the context of class imbalance), we decided to explore the 80/20 split version (which allows for training the models on a larger number of cases and, at least in theory, offers the possibility of achieving superior performance to the 70/30 split).

In an attempt to obtain the best performance of each model, we decided to optimize the models as follows:

- Logistic regression: Inverse weights were applied for the classes (to correct class imbalance) and L1 (Lasso), L2 (Ridge) and Elastic-Net-type penalties were tested (to reduce the overfitting effect and select the relevant variables); it is a method that combines the advantages of L1 (sparse regularization, variable selection) and L2 (stability).
- Random Forest: Hyperparameters were adjusted (such as the number of trees, maximum tree depth, minimum number of samples per leaf), and weights were applied to reduce minority class classification errors.
- XGBoost: Boost parameters (learning_rate, max_depth, n_estimators, subsample) and class weights were adjusted to improve performance in detecting cases with complications.

Hyperparameters for each algorithm were tuned using a grid search approach on the training set. The final parameter settings are summarized in Supplementary Table S1.

### 2.5. Comparing the Performance of Predictive Models

All models were tested comparatively (in both unoptimized and optimized variants) by applying the same evaluation procedure on an unrevised test set (hold-out technique) comprising either 20 or 30% of the initial data depending on the model split. The following indicators were used to evaluate the performance of each model:

- Overall accuracy.
- AUC ROC (area under the receiver operating characteristic curve) score—to measure the overall ability to discriminate between classes.
- Sensitivity (recall) for class 1 (ability to correctly identify patients with complications)—essential in a medical context, as the inability to identify cases that have the potential to suffer complications can have serious clinical consequences.
- Specificity (ability to correctly identify cases without complications)—clinically significant, as it confirms the possibility of safe drug administration.
- F1-score for class 1—indicates the balance between precision and sensitivity.
- Error rate—indicates the total proportion of incorrect classifications (false positive and false negative).

Following the comparative analysis, we identified the model with the best performance (optimized Random Forest 80/20), which was subsequently used to generate clinical tools with practical applicability—a risk score and an interactive HTML form that provides access to an automatic risk calculator.

### 2.6. Internal Validation

To provide a more robust estimate of model generalizability and reduce the risk of overfitting, we subsequently applied a 5-fold stratified cross-validation procedure across the entire dataset. Performance metrics (AUC, sensitivity, specificity, accuracy) were calculated as the mean across folds. To assess stability, we also performed repeated 5 × 5 CV (25 runs). In addition, we generated out-of-fold (OOF) predictions across all runs and computed a bootstrap 95% CI for AUC (B = 2000). While a nested cross-validation framework would have been more rigorous, this was not feasible in the current proof of concept due to dataset size limitations.

## 3. Results

### 3.1. General Description of the Cohorts and Distribution of the Target Variable

The analytic dataset comprised 395 treatment episodes from a single center, of which 177 (44.8%) developed at least one bevacizumab-related complication and 218 (55.2%) did not. Each row represents one treatment episode; when patients contributed multiple episodes, these were handled as independent observations after confirming no data leakage across the train–validation partitions. The primary outcome was the occurrence of any bevacizumab-related complication during the observation window.

**Figure 1:**
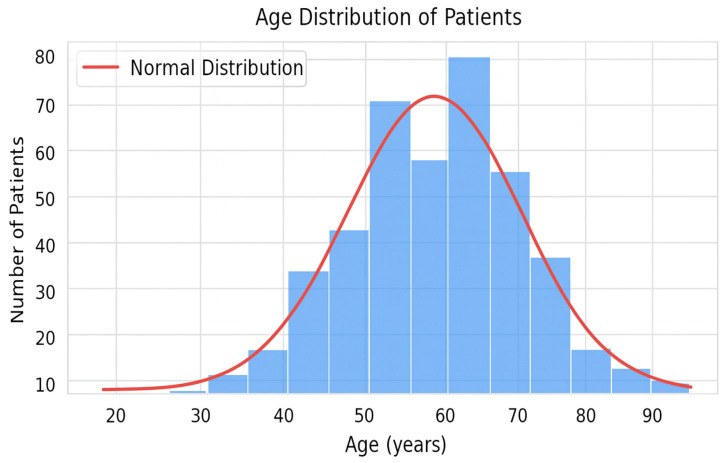
Histogram of the age of patients in the studied cohort.

The overall sample contained 130 male and 265 female patients. The patients mostly came from urban areas (341 patients—86.33%), suggesting an inequality of access to oncological medical services for the rural population. Further, 271 patients were retired at the time of treatment, 90 were employees and 34 were without insurance, benefiting from access to complex treatments through the National Oncology Program (the fact that 8.61% of the total patients belong to this group suggests that the program is functional in the IOB). Figure 1 presents an age histogram in the studied cohort. From the age histogram, it is observed that the distribution of this variable is relatively normal with a slight negative asymmetry (skewness = −0.18). A relatively normal distribution indicates that our group is representative for the statistical analysis, the age of the patients not being a bias factor. The age of the patients ranged from 22 to 88 years, with a mean age of 61.6 ± 10.88, which confirms that the majority of patients treated with Bevacizumab for solid tumors were in their sixth decade of life, which coincides with the usual age of diagnosis in oncology. The presence of young patients (under 40 years), although a minority, proves that there are advanced cases in young populations as well—these patients have certain psychosocial characteristics that must be taken into account when establishing the therapeutic plan. The fact that the two groups do not appear to be statistically different (*p* = 0.475) is important because the correlation between advanced age and patients’ comorbidities is known. The general fragility of the body induced by advancing age may, at least in theory, increase the frequency of complications associated with Bevacizumab therapy.

Table 1, Table 2 and Table 3 present the distribution of pretherapeutic variables, based on which we analyzed the global sample. We observe that most pretherapeutic variables have a relatively balanced distribution between the two groups. The only variables that seem to be more frequent in the group with complications are cardiac and thromboembolic history, chronic treatment with anticoagulants, pre-existing renal damage, low serum hemoglobin values and certain tumor characteristics (colorectal or cervical location of the primary tumor, poor/absent cellular differentiation and more aggressive histologies).

Table 4 summarizes the number, type and severity of complications occurring in our patients under Bevacizumab/biosimilar therapy.

### 3.2. Comparative Interpretation of Predictive Models’ Performances

Model optimization had heterogeneous effects on performance: while the Random Forest model benefited significantly from hyperparameter tuning (optimization resulted in an increase in AUC from 0.66 to 0.75, while maintaining a good balance between sensitivity and specificity, thus resulting in the best-performing predictive model), no notable improvements in performance metrics were observed for predictive models based on logistic regression or XGBoost. Moreover, in the case of the XGBoost 80/20 model, a reduction in performance was observed after hyperparameter tuning (although the AUC remained at approximately the same value, the sensitivity of the model decreased to 0.400).

In the course of trying to improve the models’ learning ability (especially in the context of class imbalance), we decided to explore the 80/20 split version (which allows for training the models on a larger number of cases and, at least in theory, offers the possibility of obtaining superior performance to the 70/30 split initially used). Comparing identical models trained on two different data splits provides the following conclusions: models trained on the 80/20 split generally perform better than their counterparts trained on the 70/30 split, both in terms of AUC-ROC and sensitivity, providing a better balance between learning and evaluation.

**Figure 2:**
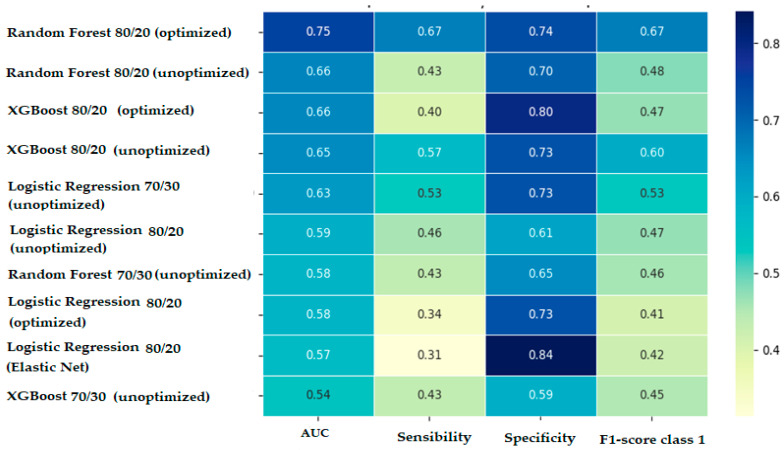
Comparative performances of trained predictive models.

The comparative performances of all trained predictive models are presented in Figure 2 in the form of a heatmap for easier visualization.

### 3.3. Performances of the Optimal Predictive Model

After training various predictive machine learning models, we found that the Random Forest model with an 80/20 split and optimized by adjusting hyperparameters and applying class weights to reduce classification errors of the minority class achieved the best prediction performance for the probability of complications associated with therapy.

Paired DeLong tests on the common validation set showed no statistically significant differences in AUC among RF, Logistic/Elastic-Net, and XGBoost (all *p* > 0.05). McNemar tests for accuracy at the 0.50 threshold were also non-significant across pairwise contrasts, supporting the interpretation that observed differences are modest; RF was, therefore, selected for downstream translation due to its balanced sensitivity/specificity and amenability to SHAP-based explanations

The overall performance of the RF 80/20 optimized model was as follows:

- Overall accuracy: 70.63%.
- Error rate: 29.37%.
- Sensitivity (recall)—detection of patients with complications: 66.67%.
- Specificity (detection of patients without complications): 73.85%.
- AUC ROC: 0.75.
- F1-score for class 1 (patients with complications): 0.67.
- Precision: 0.673.

The optimized Random Forest 80/20 model, trained on the group of 395 patients (177 presenting complications and 218 who did not develop complications), demonstrated moderate predictive performance but very well balanced between classes. Moreover, the overall accuracy of 70.63% tells us that the model manages to correctly classify 7 out of 10 patients. The recall value for the minority class of 66.67% means that the model manages to detect two out of three patients who will develop complications associated with therapy, while the specificity of 73.85% shows a satisfactory ability to exclude patients who will not develop complications (correctly detecting three out of four patients in this category). The clinical importance of these results, especially in the context of oncology practice, can be considerable, avoiding unnecessary degradation of the patient’s quality of life and interruption of systemic therapy. Such a model can be successfully used as a tool for initial triage of patients, but the error rate of 30% requires caution in its use as the sole decision-making criterion. The accuracy of 67.3% of the model (a value comparable to the sensitivity) suggests that our model is well calibrated between detecting cases and avoiding, as much as possible, false alarms. And the F1-score value of 67%, being the harmonic mean between sensitivity and precision, supports the conclusion that the optimized Random Forest 80/20 model has a satisfactory, balanced performance, without being unbalanced in a certain direction (increased precision at the expense of poor recall or vice versa). Such a model finds its clinical applicability in situations where both risks (of missing certain cases or of generating false alarms) are important. Finally, the AUC ROC value (0.75) indicates that this model has a reasonable discriminative power (well above the random threshold of 0.5) in a varied range of decision thresholds.

**Figure 3:**
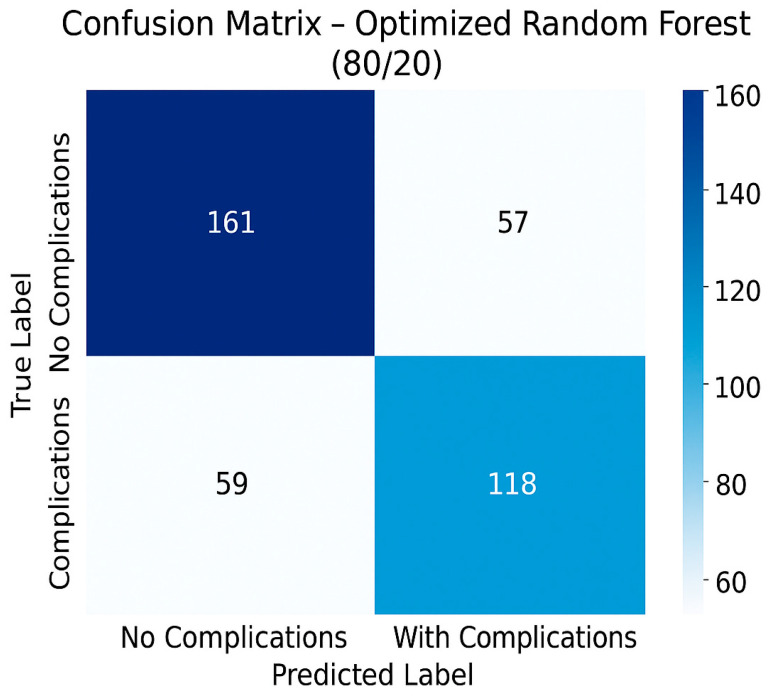
Confusion matrix of the optimized Random Forest model (80/20).

In terms of possible errors, the model records both false-negative results (patients who will develop complications but whom the model incorrectly labeled as risk free) and false-positive results (patients who will not develop complications but whom the model classifies as at risk). In the context of current oncological practice, false-negative results can have serious consequences through the lack of adequate monitoring or the absence of treatment adjustment. In contrast, false-positive results, although not inherently dangerous for the patient, can cause unnecessary anxiety, additional costs associated with the medical act generated by over-investigation and excessively cautious monitoring. In Figure 3, we present a confusion matrix of the optimized Random Forest 80/20 model, which gives us an overview of these potential errors.

Based on the above, we can conclude that the optimized Random Forest 80/20 model can be used as an automated screening tool that allows the treating physician to identify patients they intend to treat with Bevacizumab who are prone to develop complications associated with therapy, contributing to dose adjustment and individualization of monitoring protocols. This model offers the most robust predictive performance, combined with a good balance between detecting cases at risk (sensitivity) and avoiding false alarms (specificity). However, the moderate performances suggest that the model can be used strictly to complement clinical assessment and not as a substitute for it.

### 3.4. Model Validation

To assess generalizability, we complemented the initial 80/20 hold-out with internal cross-validation. In 5-fold stratified cross-validation, the optimized Random Forest model achieved an AUC of 0.62 ± 0.10, accuracy 0.60 ± 0.08, sensitivity 0.43 ± 0.11, specificity 0.73 ± 0.09, precision 0.57 ± 0.10, and F1 0.49 ± 0.10 (Supplementary Table S2). Repeating the procedure (5 × 5 cross-validation; 25 runs) yielded nearly identical estimates: AUC 0.62 ± 0.07, accuracy 0.59 ± 0.06, sensitivity 0.43 ± 0.08, specificity 0.72 ± 0.06, precision 0.56 ± 0.08, and F1 0.48 ± 0.08 (Supplementary Table S3). Out-of-fold predictions aggregated across all runs yielded a global AUC of 0.612 with a bootstrap 95% CI of 0.555–0.665 (B = 2000), overlapping the cross-validated means and supporting the stability of our estimates (Supplementary Table S4).

Class proportions were preserved across folds (positives ≈45%), indicating that the observed trade-off—higher specificity than sensitivity—reflects the model rather than sampling artifacts. This profile suggests a ‘rule-in’ orientation: positive flags are clinically meaningful for intensifying monitoring in higher-risk patients, while negative predictions should not be used to de-escalate care.

These findings are in line with this study’s proof-of-concept aim—demonstrating clinician-led feasibility on real-world data—rather than maximizing performance. Future multicenter external validation, feature enrichment, and threshold calibration are expected to improve sensitivity and overall utility.

### 3.5. Model Interpretability (SHapley Additive exPlanations—SHAP)

**Figure 4:**
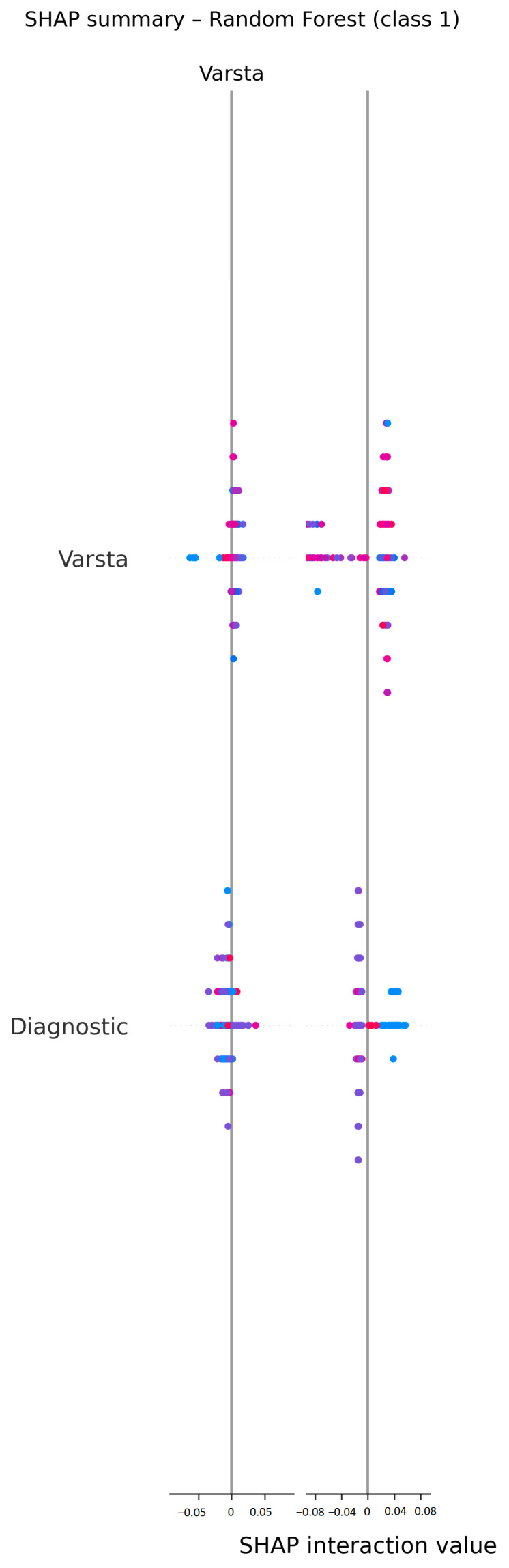
SHAP summary plot for the optimized Random Forest model. Each dot represents one patient; the x-axis shows the feature’s contribution to the predicted probability of complications (positive values increase risk). Color encodes the feature value (blue = low, red = high). Age and Diagnosis are the most influential features: higher age and specific diagnostic categories push predictions toward higher risk, whereas lower age and other categories push them downward. The modest effect sizes (|SHAP| ≲ 0.05) mirror the cross-validated performance (AUC ≈ 0.62), indicating that risk is distributed across several routine variables rather than dominated by a single predictor.

To examine how predictions were formed, we generated a SHAP summary plot for the optimized Random Forest model (Figure 4). This step increases clinical transparency and aligns with current recommendations for explainable AI in medicine [17,18,19].

Two variables concentrate most of the explanatory signal: age (Varsta) and diagnosis (Diagnostic). In the plot, each dot is one patient; the horizontal position represents the direction and magnitude of that feature’s contribution to the predicted probability of complications (positive values push the prediction toward “complication”), while color encodes the feature value (blue = low, red = high).

Overall, higher age values systematically shift predictions toward higher risk, whereas younger age shifts them downward. For diagnosis, certain diagnostic categories tend to push risk upward while others push it downward, reflecting the expected heterogeneity across tumor sites/contexts. Importantly, the absolute SHAP magnitudes are small (roughly within ±0.05), indicating no single dominant driver and aligning with the modest discrimination observed in cross-validation (AUC ≈ 0.62). In other words, risk is multi-factorial, with several routine variables contributing incrementally rather than any one predictor determining the output.

From a clinical standpoint, these explanations are plausible and transparent: older or diagnostically less favorable profiles nudge predictions upward, supporting the tool’s intended use for risk awareness and triaging of monitoring intensity rather than dictating therapy. The plot, thus, illustrates that our clinician-led model behaves in a way that is consistent with clinical intuition while remaining interpretable for non-IT users.

### 3.6. Translating the Predictive Model into Clinical Tools Applicable in Oncological Practice

**Figure 5:**
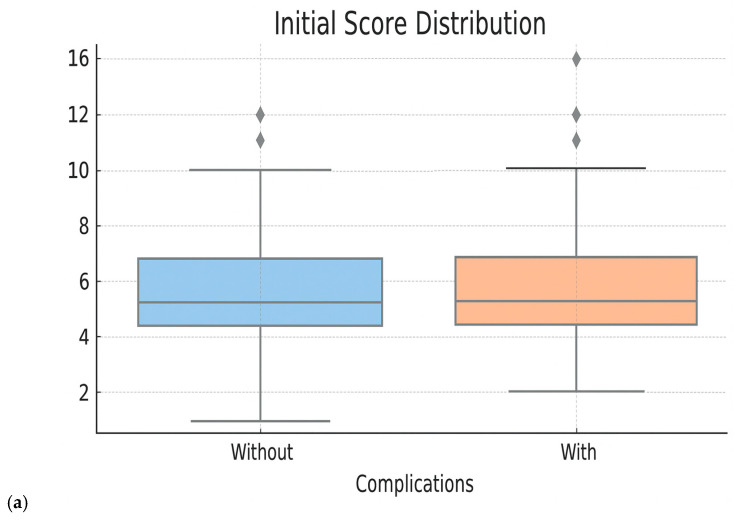

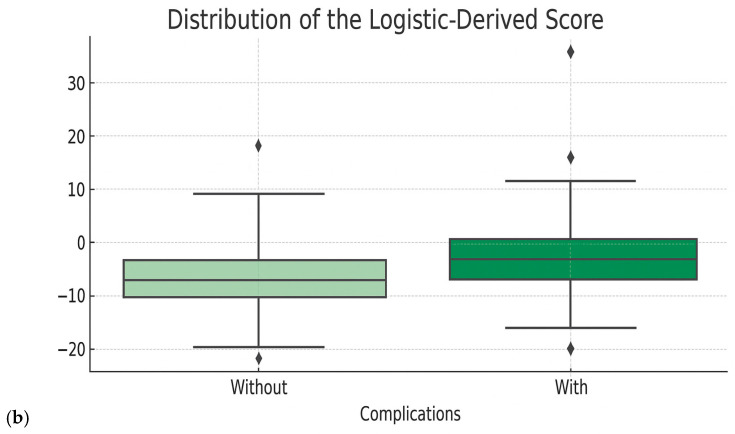
Distribution of clinical risk scores according to the occurrence of complications—comparison between the initial version (**a**) versus the logistic derived score (**b**). The Y-axis shows the raw model score (log-odds); positive values increase the estimated probability and negative values decrease it.

**Figure 6:**
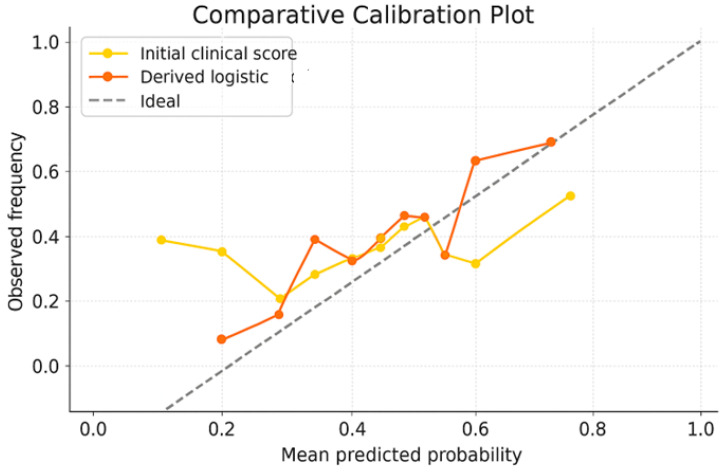
Comparing calibration plot for the logistic derivative score and the original score.

Following the comparative analysis, we identified the model with the best performance (optimized Random Forest 80/20), which was subsequently used to generate clinical tools with practical applicability—a risk score and an interactive HTML form that provides access to an automatic risk calculator. This step-by-step process consisted of the following:

1. Selection of major predictive variables: The first step in the construction of the clinical score consisted of extracting the most relevant predictive variables by analyzing the importance coefficients of the variables generated by the selected model. We were, thus, able to identify variables that the predictive model considered to have greater predictive power. Of these, those that were easily accessible in current practice, available pretherapeutically and clinically significant were retained. The list of biological parameters considered predictive included the following: hemoglobin level, platelet and leukocyte count, blood glucose, creatinine, urea and transaminase values. Clinical parameters, such as age, location of neoplastic disease, stage of disease and degree of tumor differentiation, were also introduced into the list.
2. Construction of the initial clinical score: For each selected numerical variable, binary thresholds were defined (e.g., Hg < 11 g/dL, urea ≥ 40 mg/dL), based on the distribution in the dataset and clinical expertise. Then, each category was assigned a score proportional to its relative importance in the optimized Random Forest 80/20 model. The scores were aggregated into a cumulative score, which was subsequently divided into three risk classes: low (0–2 points), intermediate (3–5 points) and high (≥6 points).
3. Testing of the initial clinical score: The score was applied retrospectively to the entire cohort to evaluate its performance in identifying patients at risk. We analyzed the distribution of patients according to score, the actual complication rates in each risk group and the statistical performances (AUC ROC, accuracy, sensitivity and specificity). The performances of the initial clinical score were as follows: overall accuracy—59%, AUC ROC—0.555, sensitivity (recall for class 1)—47.2%, specificity—60.8%, F1-score class 1—0.502. These results suggest a low capacity to triage patients exposed to Bevacizumab according to the risk of developing specific complications. Moreover, since the initial score has a discrete nature, with fixed thresholds, the premises of some limitations in the fine representation of individual risk are created. These aspects motivated us to try to optimize the score.
4. Optimization of clinical score performances: The refinement of the clinical score was performed by applying a logistic regression model, using the derived raw score as predictors. A calibrated probability for the occurrence of complications was, thus, generated, which allowed us to eliminate arbitrary thresholds and generate a continuous risk estimate and generate a new logistic-derived clinical score. The advantages of the logistic-derived score are as follows: personalized probabilities of risk occurrence, better separation of high-risk from low-risk cases, and the possibility of validation through calibration (observed vs. predicted ratio). A simplified logistic regression-based risk score was derived from the multivariate logistic model. Regression coefficients (β) were scaled and rounded to obtain integer weights, which were then summed into a total score. Cut-off points for risk stratification (low, intermediate, high) were selected based on Youden’s J index from ROC analysis.
5. Performance evaluation of the optimized score: The performances of the new score were compared with the initial score and with the Random Forest model directly through ROC curves, analysis of the score distribution and performance indicators—accuracy, sensitivity, specificity, F1-score. These evaluations showed superior performances to the initial score, comparable to those of the Random Forest model, but with the additional advantage of clinical portability and increased interpretability (Figure 5 and Figure 6 and Table 5). When analyzing the comparative distribution of scores, we find that for the initial score, no clear separation is observed (the scores of patients with and without complications often overlap), indicating a limited discrimination capacity, with a close mean between groups and a large variation. In contrast, the logistic-derived score shows a clearer separation between groups. Patients who developed complications tend to have higher probability scores, while patients without complications are concentrated in the lower part of the distribution.

Validation of the probabilities predicted by the logistic-derived score: In order to best assess whether the probabilities given by the logistic-derived score reflect the real risk of complications, but also for a better comparison with the initial score, we generated the comparative calibration plot shown in Figure 6.

The calibration plot shows the agreement between the probabilities of complications predicted by each score and their actual observed frequency. The dotted diagonal line represents the ideal scenario, in which there is perfect agreement (the model accurately predicts the risk in all cases). In Figure 6, we observe that the logistic derivative score curve approaches the ideal line, which indicates both increased discrimination power (the model separates patients with and without complications quite well) and the fact that the score correctly estimates the probability of risk. Minor deviations from the ideal line in certain areas can be attributed to the natural imbalance existing in the dataset, but this does not significantly affect the overall performance. Therefore, we concluded that the optimized score has a robust calibrated prediction capacity, which makes it suitable for clinical applications.

### 3.7. Building an Interactive Form Applicable in Oncologic Practice

Based on the logistic-derived score, we built a digital HTML form with a responsive design (which allows for automatic adaptation to the electronic device from which it is accessed—mobile, tablet, laptop), accessible and offline, in which the doctor can tick the presence of risk factors, and the system automatically returns the cumulative score, risk class and estimated percentage risk. The form is easy to use and has the potential to be integrated into oncological practice, offering treating physicians a tool for pretherapeutic stratification of the risk of developing therapeutic complications. Table 6 and Table 7 present the final version of the logistic-derived score (variables, categories and scores awarded) and the clinical risk thresholds.

Supplementary Material File S1 consists of an interactive HTML form of the risk calculator based on the clinical risk score, proving its functionality.

## 4. Discussion

In the continuous effort to ameliorate post-therapeutic results [20,21,22], the oncology landscape is rapidly evolving, with a huge influx of real-time clinical, paraclinical, imaging, genetic and molecular data that exceed the capacity of the standard decision-making processes of the human physician. Advanced AI tools can synthesize this complexity and support personalized treatment decisions, without replacing clinical experience, normal interaction between patient and physician or evidence-based international guidelines. Various forms of AI tools have been employed in developing CDSSs that can be integrated into oncological practice with ease and playing multiple roles:

- Machine learning algorithms (Random Forest, Gradient Boosting such as XGBoost, Support Vector Machines, logistic regression)—used for developing predictive models based on historical data. They work by identifying patterns in a dataset and applying them to new cases, providing quantifiable and reproducible results. Possible applications include prediction of risk of toxicity/adverse effects, prognostic stratification of patients or classifying therapeutic response to various agents. One such example is xDECIDE, which is a web-based platform that combines AI power with human clinical expertise and offers personalized oncological therapeutic recommendations. Studies have proven that this platform has real impact on decision making; changes in the initial treatment plan proposed by the clinician were recorded after using the platform [23,24].
- Deep Learning is an extension of machine learning, which employes complex neural networks with multiple layers to learn highly abstract representations of data. These models, such as CNN (Convolutional Neural Networks) or LSTM (Long Short-Term Memory), are capable of analyzing medical images (such as CT, MRI, histopathology) or time-series data (such as evolution of biological parameters). Deep Learning is used for the automatic recognition of anomalies and interpretation of sequential data. Deep Learning can be used for automatic recognition of lesions on CT scans and MRI or for early detection of toxicity signs based on serial analyses. An example of real applications of Deep Learning-based CDSS is DeepMind Health (Google), which employs Complementarity-Driven Deferral to Clinical Workflow (CoDoC) for screening of breast lesions. Compared to double reading with arbitration, the predictive model reduced false-positive rates by 25% and reduced clinician workload by 66% [25]. Clinical trials on the deployment of Deep Learning CDSSs in healthcare are underway with encouraging results [26].
- Natural Language Processing (NLP)—allows AI to “understand” and process human written speak, thus being able to extract valuable information from unstructured medical records (charts, clinical or trial notes, guidelines) and transforming the data into structured data usable for CDSS development. NLP tools, such as Named Entity Recognition (NER), text summarization or Embedding models (e.g., Word2Vec, BERT), can be used for automatic extraction of data from medical records, identifying eligible patients for clinical trials and generation of guideline-concordant therapeutic recommendations [27,28,29,30,31,32].
- Large Language Models (LLMs)—recent developments like ChatGPT, Flamingo, PaLI or Gemini led to the emergence of conversational AIs capable of coherent text generation and real-time synthetization of data from multiple sources. LLMs can be incorporated into interactive interfaces where the clinician can interact with the system, ask questions, demand explanations or viable alternatives for the proposed clinical/therapeutic decision. LLMs can also be used for automatic generation of documentation, thus reducing clinician workload. AMIE and MedGemini (medical LLMs created by Google DeepMind in 2025) have managed to generate complex oncologic therapy plans in more than 90% of the simulated cases and surpass human experts in text summarization and document generation [33,34,35,36].
- Reinforcement Learning (RL)—a lesser-known but very promising type of AI, which learns through interactions and rewards. It can be used in scenarios in which the therapeutic decision needs to be dynamically adaptative (e.g., adjusting doses based on individual patients’ response and reaction) [37,38,39]. This type of AI resembles the way a physician refines his clinical intuition after gaining experience based on the results of previous decisions.
- In order for AI to be truly useful in clinical practice, it needs to be transparent, and the variable which formed the bases for a decision needs to be easily explained to the human doctor/patient. Higher transparency will ensure better adoption of AI technologies into clinical practice. Explainable AI (XAI), through technologies such as LIME or SHAP, has precisely this role [40,41].
- At the heart of all these technologies is Data Engineering—the process of collecting, cleaning, and shaping medical data. Without this step, no AI model will work properly. Libraries like Pandas, NumPy or TensorFlow Data are used to convert raw data into formats that can be used by algorithms.
- Finally, in the context of multicenter collaboration, Federated Learning offers an ethical and safe solution to train AI models on distributed databases (data from multiple medical centers) without sharing patient information between institutions (e.g., Flower, NVIDIA Clara, TensorFlow Federated). In this way, hospitals can contribute to shared models while respecting data privacy under GDPR or HIPAA regulations.

These types of AI are not competing but complementary to each other and to human clinical expertise. Each adds an essential piece to the complex puzzle of assisted medical decision making.

### 4.1. Related Work and State-of-the-Art (SOTA) Approaches

Recent frameworks, such as the Yonsei Cancer Data Library, demonstrate how continuous multimodal data integration empowers evidence-based guidance across over 170,000 cancer cases [42]. Combined, multiple AI tools enable the transition from standard guidelines to personalized real-time assistance, supporting the oncologist in their mission to provide safe, effective and tailored care for each patient [2,4,5,9,43,44]. In Table 8, we present some of the more prominent usage of AI-CDSS in oncology.

**Figure 7:**
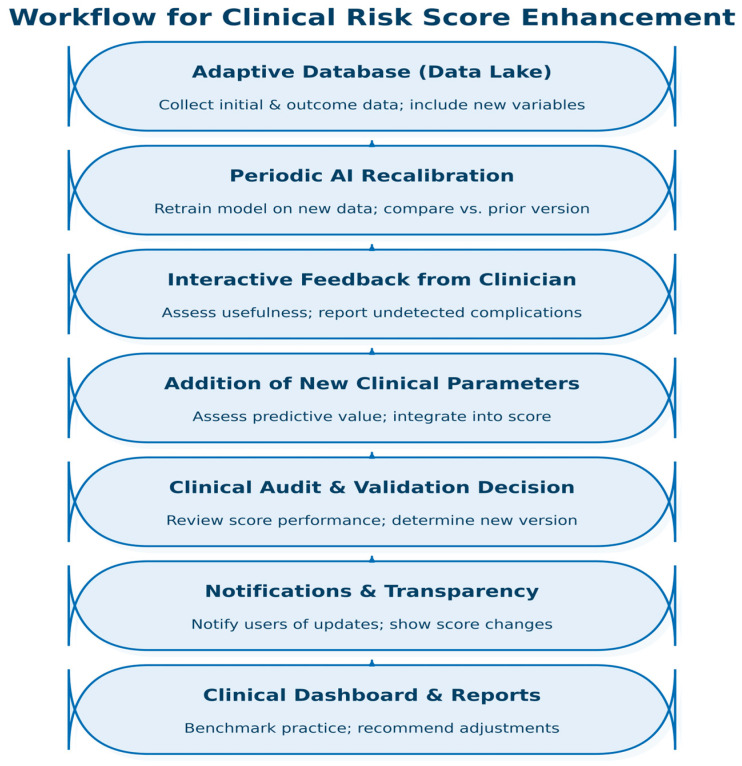
Proposed structured workflow for continuous refinement of an AI-CDSS-based risk score used in oncology clinical practice.

While these SOTA systems illustrate the remarkable potential of AI in medicine, our work positions itself differently, as a clinician-led proof of concept, showing that accessible AI tools can already be integrated into everyday oncology practice, even outside large research consortia or IT-driven environments. The distinctive feature of this study is that it was designed and conducted entirely by clinicians, without formal training in computational sciences or artificial intelligence. This underscores the important shift in the field: AI-based technologies have become sufficiently accessible and intuitive to be employed by non-specialists using real-world clinical data (especially once clinicians surpass their initial reticence and embrace the new technologies). However, we recognize that collaboration with AI experts is crucial to further model enhancement, optimization, interpretability, validation and clinical implementation. Integration of AI-CDSS into oncological practice offers a transformative opportunity, but to ensure clinical relevance and performance over time, a dynamic, feed-back-driven continuous development approach must be considered and implemented. In Figure 7, we propose a structured workflow, which allows for continuous refinement of AI-CDSS-based clinical scores for predicting complications associated with oncologic agents.

In order for it to be possible to integrate new data and improve the model over time, first, we need to establish an adaptive clinical data repository (data lake) that captures both baseline and outcome variables from real-world clinical practice. The database needs to be designed for expansion with the inclusion of new key clinical parameters as they emerge from evolving research or clinical observation. As recommended by frameworks for AI-CDSS, incoming real-world clinical data fed into adaptive databases are the bases for incremental learning and retraining of predictive models [54,55]. By design, database architecture should allow for modular data expansion, thus allowing the incorporation of new emerging biomarkers or clinical variables without disrupting the existing workflows [4,23,42]. An upgradable AI-CDSS driven by clinician feed-back, real-life data accrual and periodic model retraining offers a scalable and ethical approach to maintaining the relevance of the tool. This dynamic framework ensures that the CDSS does not become outdated but rather evolves parallel to medical evidence, therapeutic innovations and patient-specific complexity.

### 4.2. Study Strengths, Limitations and Future Directions of Research

This study has several notable strengths. First, the model is built exclusively on real-world, prospectively collected clinical data and uses only pretherapeutic variables, thus enabling early risk stratification before the actual initiation of Bevacizumab/biosimilars therapy. A key innovation is that the entire model development was carried out by clinicians with no formal computational training, underscoring the growing accessibility and usability of AI tools in daily medical practice. Moreover, the model was translated into intuitive, offline, HTML interactive tools, with immediate deployment capability, designed for direct clinical use. And, while the predictors identified by the model (e.g., age, anemia, renal function, tumor differentiation) are well known to oncologists, the added value of this tool lies in its ability to synthesize multiple variables into a single, individualized risk estimate that can aid in triaging and shared decision making, especially in borderline or frail patients. Special attention is given to model transparency, simplicity and clinical relevance, bridging the gap between theoretic modeling and clinical practice. As such, this study does not position itself as a technical AI project but rather as a clinician-led proof of concept, demonstrating how AI tools can now be realistically implemented in oncology care. At the same time, we emphasize the value of interdisciplinary collaboration with AI experts to further refine model performance, interpretability and clinical integration. This combination of accessibility, clinical grounding and translational focus contributes to the growing body of evidence that AI can support (and not replace) medical expertise in personalizing cancer care.

The most important limitations of our study are as follows:

- The most significant limitation of our study is the fact that our risk score is not developed using an adaptive database, and, as a result, our predictive models cannot be retrained once new clinical data become available. This was done to highlight that even in the absence of a trained AI expert, AI tools have become so readily available and intuitive that medical professionals can employ them in their current research and practice. However, collaboration with AI experts is highly recommended for optimal results.
- Relatively small and unbalanced sample size: The relatively modest size of the dataset and the unequal distribution between patients in the group with and without complications (unbalanced classes) may limit the generalizability of the predictive models that we trained. While 395 treatment episodes were included, we acknowledge that the sample size may still limit the statistical power of the model, particularly in edge-case scenarios. This reinforces the need for further multicenter validation.
- The data come from a single medical unit, which introduces an institutional selection bias, which reduces the external validity of the results obtained and increases the risk of overfitting.
- Absence of potentially relevant variables: Some clinical and biological information that could have predictive value for the occurrence of complications (such as concomitant treatments, detailed ECOG score, immunohistochemical factors, the presence of oncogenic mutations and mutation repair deficiencies) was available for a very small number of patients, which made them statistically unusable.
- The final risk score has not been validated on an external dataset, nor has it been prospectively tested in clinical practice to prove its reliability.

These limitations also lead to future directions for further research:

- Implementing a data-lake adaptive system will allow for continuous refinement of clinical tools developed and maintaining clinical relevance.
- Increasing the sample size by introducing data from other medical centers (multicenter study) could strengthen the robustness and generalizability of the predictive models.
- Prospective validation of the score on an external sample is necessary to test its performance and utility in bedside decision making. The current study represents a single-center proof of concept. Future work should aim to externally validate the model on independent cohorts, ideally through multicentric collaborations.
- Intense collaboration with IT experts would mainstream the process of developing better AI tools.
- Future versions of the predictive model could include other variables (such as immunohistochemical and genetic markers or other imaging, clinical and therapeutic data) to increase the accuracy of predictions
- The development of offline digital applications such as the one already created paves the way for personalized decision support tools, easy to use for clinicians.

## 5. Conclusions

The present study has proven the possibility of constructing and internally validating a clinical score that is easy to introduce into current oncological practice, using predictive models based on machine learning algorithms, capable of estimating the risk of patients developing a complication associated with Bevacizumab or biosimilar therapy based on the data usually available pretherapeutically. Through a rigorous, staged approach, we obtained a clinical tool with practical value that is very easy to implement. Moreover, the transformation of the score into a digital HTML form with a responsive design (which allows automatic adaptation to the electronic device from which it is accessed—mobile, tablet, laptop), accessible and offline, facilitates clinical implementation and makes it directly usable by physicians within the therapeutic decision-making process. The clinical risk score facilitates good risk stratification and allows for personalized management of the oncology patient treated with Bevacizumab or biosimilars. Our tool is not designed to dictate therapeutic decisions but rather to support risk awareness and individualization of monitoring strategies. Even a somewhat modest predictive performance (70% accuracy with balanced sensitivity/specificity) can help avoid underestimating risks in complex patients and facilitate shared decision making between doctor and patient.

The present study is a clinician-led proof of concept aimed at demonstrating that contemporary AI tools can be safely explored by physicians with transparent outputs, not an IT state-of-the-art benchmark. This study is single center and lacks external validation; discrimination is modest, with a specificity-dominant profile (rule-in orientation). Although not meant as a substitute for clinical evaluation and paraclinical monitoring of oncologic patients, these tools may in fact provide a therapeutic decision aid for physicians and adjust the risk/benefit ratio of targeted therapies, thus potentially being the next step towards truly personalized medicine.

In sum, the present work is not intended to replace clinicians or IT experts but to reduce apprehension and bridge disciplines by showing that user-friendly AI can already support risk awareness and monitoring triage in routine oncology. Superior models will require—and we explicitly invite—multidisciplinary collaboration and external validation.

## Figures and Tables

**Table 1:** Distribution of pretherapeutic socio-demographic and medical variables.

Variable	Whole Sample	Study Group (with Complications)	Witness Group (Without Complications)	*p*-Value *
**Sex**				0.259
Male	130 (32.91%)	66	64	
Female	265 (67.09%)	152	113	
**Age**				0.475
Extremes 22–88	61.6 ± 10.88	61 ± 11.0	62.4 ± 10.7	
**Residence environment**				0.167
Urban area	341 (86.33%)	183	158	
Rural Area	54 (13.67%)	35	19	
**Blood type**				0.987
A	164 (41.52%)	92	72	
O	139 (35.19%)	76	63	
B	58 (14.68%)	32	26	
AB	34 (8.61)	18	16	
**Rhesus factor**				0.667
Rh-positive	321 (81.27%)	175	146	
Rh-negative	74 (18.73%)	43	31	
**Comorbidities**				
Hypertension	147 (37.22%)	73 (33.5%)	74 (41.8%)	0.1103
Ischemic heart disease	29 (7.34%)	10 (4.6%)	19 (10.7%)	**0.0327**
Cardiac insufficiency	33 (8.35%)	12 (5.5%)	21 (11.9%)	**0.0367**
Valvopathies	62 (15.70%)	26 (11.9%)	36 (20.3%)	**0.0318**
Arrythmias	32 (8.10%)	15 (6.9%)	17 (9.6%)	0.4230
Stroke/TIA	3 (0.76%)	1 (0.5%)	2 (1.1%)	0.8560
PTE/DVT	20 (5.06%)	6 (2.8%)	14 (7.9%)	**0.0362**
Diabetes	44 (11.14%)	18 (8.3%)	26 (14.7%)	0.0629
Chronic lung disease	20 (5.06%)	9 (4.1%)	11 (6.2%)	0.4779
Chronic renal disease	10 (2.53%)	1 (0.5%)	9 (5.1%)	**0.0096**
Chronic hepatitis or cirrhosis	13 (3.29%)	6 (2.8%)	7 (4.0%)	0.7020
Previous cancer	34 (8.61%)	21 (9.6%)	13 (7.3%)	0.5313
Psychiatric disorders	15 (3.80%)	6 (2.8%)	9 (5.1%)	0.3465
Recurrent urinary infections	15 (3.80%)	11 (5.0%)	4 (2.3%)	0.2396
Previous surgery	288 (72.91%)	158 (72.5%)	130 (73.4%)	0.9190
**Chronic/previous treatments**				
Oral Anticoagulants	29 (7.34%)	9 (4.1%)	20 (11.3%)	**0.0116**
Oral Antidiabetics	20 (5.06%)	7 (3.2%)	13 (7.3%)	0.1025
Insulin	4 (1.01%)	1 (0.5%)	3 (1.7%)	0.4746
Bevacizumab	55 (13.92%)	28 (12.8%)	27 (15.3%)	0.5878
Neoadjuvant Immunotherapy	94 (23.80%)	44 (20.2%)	50 (28.2%)	0.0796
Neoadjuvant Chemotherapy	234 (59.24%)	134 (61.5%)	100 (56.5%)	0.3698
Neoadjuvant Radiotherapy	92 (23.29%)	47 (21.6%)	45 (25.4%)	0.4331
Hormonotherapy	5 (1.27%)	2 (0.9%)	3 (1.7%)	0.8143
**Nutritional status**				0.2531
Normal weight	257 (65.06%)	148 (67.9%)	109 (61.6%)	
Overweight/Obesity	73 (18.48%)	40 (18.3%)	33 (18.6%)	
Underweight/Cachexia	65 (16.46%)	30 (13.8%)	35 (19.8%)	

Abbreviations used: TIA—transient ischemic attack, PTE—pulmonary thromboembolism, DVT—deep venous thrombosis, *—*p*-value is calculated using the Chi-square test, *t*-test or Mann–Whitney U (Wilcoxon rank-sum) test depending on the type of variable.

**Table 2:** Distribution of pretherapeutic variables specific to the neoplastic disease.

Variable	Whole Sample	Study Group (with Complications)	Witness Group (Without Complications)	*p*-Value *
**Type of cancer**				**0.0002**
Colo-rectal	209 (52.91%)	104 (47.7%)	105 (59.3%)	
Ovarian/tubal/primary peritoneal	102 (25.82%)	70 (32.1%)	32 (18.1%)	
Cervical	40 (10.13%)	16 (7.3%)	24 (13.6%)	
Breast	28 (7.09%)	22 (10.1%)	6 (3.4%)	
Pulmonary	7 (1.77%)	4 (1.8%)	3 (1.7%)	
Others	9 (2.28%)	2 (0.9%)	7 (4.0%)	
**Histopathology**				**0.0013**
Adenocarcinoma	206 (52.15%)	104 (47.7%)	102 (57.6%)	
Serous carcinoma	93 (23.54%)	63 (28.9%)	30 (16.9%)	
Squamous carcinoma	33 (8.35%)	13 (6.0%)	20 (11.3%)	
Invasive ductal/lobular carcinoma	25 (6.33%)	20 (9.2%)	5 (2.8%)	
Mucinous adenocarcinoma	23 (5.82%)	9 (4.1%)	14 (7.9%)	
Others	15 (3.80%)	9 (4.1%)	6 (3.4%)	
**Differentiation degree**				**0.0065**
Poorly differentiated (G3)	144 (36.46%)	92 (42.2%)	53 (29.9%)	
Moderate differentiated (G2)	227 (57.47%)	110 (50.5%)	117 (66.1%)	
Well differentiated (G1)	24 (6.07%)	16 (7.3%)	8 (4.52%)	
**Perineural invasion**	75 (18.99%)	40 (18.3%)	35 (19.8%)	0.7049
**Lymphovascular invasion**	117 (29.62%)	64 (29.4%)	53 (29.9%)	0.9877
**Peritumoral lymphocyte infiltrate**	79 (20.00%)	36 (16.5%)	43 (24.3%)	0.1473
**Stage**				0.3306
IV	316 (80.00%)	168 (77.1%)	148 (83.6%)	
III	74 (18.73%)	46 (21.1%)	28 (15.8%)	
II	4 (1.01%)	3 (1.4%)	1 (0.6%)	
I	1 (0.25)	1 (0.5%)	0 (0.0%)	
**Metastasis**				
Peritoneale	142 (35.95%)	81 (37.2%)	61 (34.5%)	0.6533
Bone	25 (6.33%)	14 (6.4%)	11 (6.2%)	1.0000
Hepatic	169 (42.78%)	89 (40.8%)	82 (46.3%)	0.238
Pulmonary	95 (24.05%)	53 (24.3%)	42 (23.7%)	0.9869
Lymphatic	46 (11.65%)	28 (12.8%)	18 (10.2%)	0.5052
Cerebral	8 (2.03%)	4 (1.8%)	4 (2.3%)	1.0000
Others	15 (3.80%)	8 (3.7%)	7 (4.0%)	1.0000
**Type of disease**				0.1061
Metastatic	314 (79.49%)	170 (78.0%)	144 (81.4%)	
Locally advanced/Unresectable	52 (13.16%)	35 (16.1%)	17 (9.6%)	
Recurrent	29 (7.34%)	13 (6.0%)	16 (9.0%)	
**Bevacizumab/biosimilar administration**				0.3935
First line	180 (45.57%)	95 (43.6%)	85 (48.0%)	
Second line	146 (36.96)	84 (38.5%)	62 (35.0%)	
Subsequent therapeutic lines	54 (13.67%)	28 (12.8%)	26 (14.7%)	
Maintenance	15 (3.80%)	11 (5.0%)	4 (2.3%)	

* *p*-value is calculated using the Chi-square test for categorical variables.

**Table 3:** Distribution of pretherapeutic paraclinical variables.

Variable	Study Group (with Complications)	Witness Group (Without Complications)	*p*-Value *
Hemoglobin	11.84 ± 1.74	12.38 ± 1.56	**0.0009**
Leucocytes	7.44 ± 2.70	7.07 ± 2.66	0.1378
Thrombocytes	286.110 ± 108.420	272.790 ± 111.240	0.2500
Glycemia	101.83 ± 26.25	103.79 ± 27.01	0.3049
Creatinine	0.77 ± 0.21	0.82 ± 0.25	0.0845
Urea	32.16 ± 13.29	34.55 ± 15.48	0.1354
TGO	23.67 ± 13.30	26.38 ± 23.24	0.8259
TGP	22.29 ± 13.29	26.04 ± 38.48	0.5718
GGT	67.38 ± 94.07	75.01 ± 106.16	0.0773
Ca^2+^	9.35 ± 0.59	9.30 ± 0.57	0.1655
Coagulation modifications	29 (13.3%)	39 (22.0%)	**0.0314**
Tumoral markers alterations	74 (33.9%)	72 (40.7%)	0.2027

* *p*-value is calculated using the Chi-square test for binary categorical variables and the Mann–Whitney U (Wilcoxon rank-sum) test for numerical variables.

**Table 4:** Number, type and severity of Bevacizumab/biosimilars related complications in study group.

Type of Complications	Low-Medium Severity (Total Number of Cases = 245)	High Severity (Total Number of Cases = 60 *)	Total
**Septic/Infectious complications**	**35**	**14**	**49**
Sepsis	2	5	7
Abscess	7	3	10
Urinary infection	11	2	13
Wound complications	4	0	4
Pneumonia	1	2	3
Others	10	2	12
**Cardiovascular complications**	**51**	**7**	**58**
Thromboembolic events	12	1	13
Hemorrhagic events (other than digestive)	8	2	10
Arrythmia	1	0	1
Arterial hypertension	30	4	34
**Gastro-intestinal complications**	**34**	**14**	**48**
Digestive hemorrhage	4	2	6
Ileus	2	0	2
Fistulas/perforative complications	3	9	12
Abdominal pain	6	1	7
Gastritis	1	0	1
Stomatitis	3	0	3
Nausea/vomiting	10	0	10
Diarrhea	5	2	7
**Genito-urinary complications**	**29**	**13**	**42**
Proteinuria	29	4	33
Renal insufficiency	0	7	7
Genito-urinary fistula	0	2	2
**Hematologic complications**	**72**	**9**	**81**
Anemia	34	0	34
Leucopenia	18	6	24
Thrombocytopenia	18	2	20
Neutropenia	2	0	0
Pancytopenia	0	1	1
**Others**	**24**	**3**	**27**
Headache/Migraine	5	1	6
Neuropathies	6	0	6
Rhinitis	1	0	1
Dyspnea	3	2	5
Dehydration	3	0	3
Anorexia	1	0	1
Cutaneous complications	5	0	5

* the total number of patients with severe therapy-related complications was 56—the difference between 56 and the total number of severe complications is explained by the fact that some patients developed more than one severe complication, while others, although having adverse reactions, none of those were classified as severe by their oncologist.

**Table 5:** Performance metrics of the logistic derivative score versus the original score.

Performance Metrics	Initial Clinical Score	Logistic Derived Score
AUC ROC	0.555	0.720
Accuracy	0.590	0.680
Sensitivity (Recall)	0.472	0.642
Specificity	0.608	0.698
F1-Score	0.502	0.662

**Table 6:** Logistic-derived score variables and points awarded for each category.

Variable	Category and Points Awarded for Each Category
Age	<65 years—0 points ≥65 years—1 point
Serum Urea	<40 mg/dL—0 points ≥40 mg/dL—1 point
Leucocytes	<10.000/mmc—0 points ≥10.000/mmc—1 point
Hemoglobin	≥10 g/dL—0 points <10 g/dL—1 point
Transaminases (TGO/TGP)	<40 U/L—0 points ≥40 U/L—1 point
Creatinine	<1.5 mg/dL—0 points ≥1.5 mg/dL—1 point
Stage	Stage I–II—0 points Stage III–IV—1 point
Differentiation degree	G1–G2—0 points G3—1 point
Lymphovascular invasion	Absent—0 points Present—1 point
Type of cancer	Breast, Ovarian, Cervical—0 points Colo-rectal, Pulmonary, Others—1 point

**Table 7:** Clinical risk thresholds of the logistic-derived score.

Points	Risk Group	Probability of Complications
0–3 points	Low risk	<25%
4–6 points	Intermediate risk	25–60%
7–10 points	High risk	>60%

**Table 8:** AI-CDSS-based tools developed for oncology—related work and state-of-the-art (SOTA) approaches.

CDSS/Test	Oncology Domain	AI Type	Key Results
Yonsei Cancer Data Library [42]	Multiple cancer types	Multimodal Deep Learning (Electronic Health Record + Genomic data)	Personalized care for >170K patients; streamlined workflow
AI for Trial Eligibility [45]	Breast cancer	NLP + Rule-based Screening	Higher sensitivity and specificity vs manual screening
MammaPrint [46,47,48]	Breast cancer	Genomic Risk Classifier (ML-based)	Avoids chemotherapy in low-risk patients (MINDACT validation)
DecisionDx-UM [49,50]	Uveal melanoma	Gene Expression Classifier (ML-based)	Accurate metastasis risk classification; accepted standard of care
VeriStrat [51,52]	NSCLC	Proteomic Classifier (ML-based)	Predicts response to EGFR inhibitors like erlotinib
LLM Agents [33,53]	Oncology simulations	LLM + Retrieval-Augmented Generation	>91% accuracy in simulated oncologic decisions with explainability

Abbreviation: CDSS—clinical decision support system; AI—artificial intelligence; NLP—Natural Language Processing; ML—Machine Learning; LLM—Large Language Model; NSCLC—Non-Small Cell Lung Cancer; EGFR—Epidermal Growth Factor Receptor; MINDACT—Microarray In Node-negative and 1 to 3 positive lymph node Disease may Avoid ChemoTherapy (clinical trial).

## Data Availability

The datasets presented in this article are not readily available because the data supporting the findings of this study are not publicly available due to institutional restrictions. Requests to access the datasets should be directed to corresponding author V.R.

## Supplementary Materials

The following supporting information can be downloaded at: https://www.mdpi.com/article/10.3390/diagnostics15182391/s1, Supplementary Table S1: Final hyperparameter settings for model optimization; Supplementary Table S2: Cross validation performance; Supplementary Table S3: Repeated CV Performance; Supplementary Table S4: OOF Bootstrap AUC; Supplementary File S1: Interactive HTML form.

## Abbreviations

The following abbreviations are used in this manuscript:

AI	Artificial Intelligence
AI-CDSS	Artificial Intelligence-based Clinical Decision Support System
AMIE	Articulate Medical Intelligence Explorer
AUC	Area Under the Curve
BERT	Bidirectional Encoder Representations from Transformers
CDSS	Clinical Decision Support System
CNN	Convolutional Neural Network
CT	Computed Tomography
DVT	Deep Vein Thrombosis
ECOG	Eastern Cooperative Oncology Group (performance status score)
EGFR	Epidermal Growth Factor Receptor
EHR	Electronic Health Record
F1	F1-Score (harmonic mean of precision and recall)
GDPR	General Data Protection Regulation
GGT	Gamma-Glutamyl Transferase
HIPAA	Health Insurance Portability and Accountability Act
HTML	HyperText Markup Language
LIME	Local Interpretable Model-agnostic Explanations
LLM	Large Language Model
LSTM	Long Short-Term Memory
MINDACT	Microarray In Node-negative and 1 to 3 positive lymph node Disease may Avoid ChemoTherapy
ML	Machine Learning
MRI	Magnetic Resonance Imaging
NER	Named Entity Recognition
NLP	Natural Language Processing
NSCLC	Non-Small Cell Lung Cancer
