# Supplementary material for: Using Artificial Intelligence to Develop Clinical Decision Support Systems—The Evolving Road of Personalized Oncologic Therapy
Source: Diagnostics (Basel). 2025 Sep 19;15(18):2391. doi: 10.3390/diagnostics15182391 (PMC12468058; doi:10.3390/diagnostics15182391)
Supplement: Supplementary file 1 [file diagnostics-15-02391-s001.zip › Supplementary File S1 - Interactive HTML form.html]

Clinical Score Calculator – Risk of Complications Associated with Bevacizumab Therapy


# Clinical Score Calculator – Risk of Complications Associated with Bevacizumab Therapy

Type of Cancer
 Breast Cancer
 Cervical Cancer
 Ovarian/Peritoneal Cancer
 Colorectal Cancer
 Lung Cancer
 Other

Cancer Stage
 Stage I–II
 Stage III–IV

Histological Grade
 G1–G2
 G3

Lymphovascular Invasion
 Absent
 Present

Biological Parameters
 Age ≥ 65 years
 Hemoglobin < 10 g/dl
 Leukocytes ≥ 10,000/mmc
 Transaminases ≥ 40 U/l
 Creatinine ≥ 1.5 mg/dl
 Urea ≥ 40 mg/dl
Calculate Score
