# Supplementary material for: Using Artificial Intelligence to Develop Clinical Decision Support Systems—The Evolving Road of Personalized Oncologic Therapy
Source: Diagnostics (Basel). 2025 Sep 19;15(18):2391. doi: 10.3390/diagnostics15182391 (PMC12468058; doi:10.3390/diagnostics15182391)
Supplement: Supplementary file 1 [file diagnostics-15-02391-s001.zip › Supplementary Table S1 - Final hyperparameter settings for model optimization.pdf]

Model	Hyperparameter	Search range / options	Final selected value
**Random Forest**	Number of estimators (trees)	100 – 1000	500
	Maximum depth	3 – 15	10
	Minimum samples per split	2 – 10	4
	Class weight	Balanced / None	Balanced
	Criterion	Gini / Entropy	Gini
**Logistic Regression**	Regularization penalty	L1, L2	L2
	Regularization strength (C)	0.01 – 10	1.0
	Solver	liblinear, saga	liblinear
**XGBoost**	Learning rate (eta)	0.01 – 0.2	0.05
	Maximum depth	3 – 10	6
	Subsample ratio	0.5 – 1.0	0.8
	Colsample_bytree	0.5 – 1.0	0.8
	Number of estimators	100 – 1000	300
